# Influence of Physical and Musculoskeletal Factors on Occupational Injuries and Accidents in Korean Workers Based on Gender and Company Size

**DOI:** 10.3390/ijerph16030345

**Published:** 2019-01-26

**Authors:** Sang Jin Park, Myunghwan Jung, Joo Hyun Sung

**Affiliations:** 1 University of Soonchunhyang, College of Medicine, Soonchunhyang University Hospital Cheonan, Department of Occupational and Environmental Medicine, 31, Suncheonhyang 6-gil, Dongnam-gu, Cheonan-si, Chungcheongnam-do 31151, Korea; oemdrsjpark@gmail.com; 2 Gyeongsang National University, College of Medicine, Research Institute of Life Sciences, Department of Microbiology, 15, Jinju-daero 816beon-gil, Jinju-si, Gyeongsangnam-do 52727, Korea; mjung@gnu.ac.kr; 3 Gyeongsang National University, College of Medicine, Gyeongsang National University Changwon Hospital, Institute of Health Sciences, Department of Occupational and Environmental Medicine, 15, Jinju-daero 816beon-gil, Jinju-si, Gyeongsangnam-do 52727, Korea

**Keywords:** musculoskeletal factors, occupational accident, occupational injury, physical factors, workplace size

## Abstract

Though the overall incidence of occupational injuries and accidents has decreased, they continue to happen. Many associated factors are known and managing them with limited resources is difficult. This study evaluates related risk factors and prioritizes their management for reducing occupational injuries and accidents at the workplace. We used data from the 4th Korean Working Condition Survey conducted by the Korea Occupational Safety Health Research Institute from June to September 2014. A total of 14,381 persons (9776 men, 4605 women) were selected; t-test, chi-square test, and logistic regression analyses were performed to analyze data. The influence of physical (vibration, noise, abnormal temperature) and musculoskeletal (awkward posture, handling of heavy objects, repetitive tasks) factors was compared according to gender and company size. The risk of occupational injuries and accidents among men was related to musculoskeletal factors and vibration in companies with “under 50” employees and with awkward posture, vibration, and noise in companies with “50 or above” employees; however, among women in companies with “under 50” employees, it was associated with only vibration. Although we evaluated only a few risk factors, prioritizing them based on gender and company size has provided new valuable information.

## 1. Introduction

Occupational injuries and accidents cause psychological, social, and economic problems for the affected employee [1]. Although they have direct consequences on the families, coworkers, and employers [2], they also indirectly increase the economic burden on society through medical, rehabilitation, and compensation costs [3,4]. As a result of the continuous efforts made to prevent occupational injuries and accidents, their incidence has declined, even though they continue to occur in South Korea and other countries. According to Eurostat, the incidence rate of fatal injuries per 100,000 employees in the EU had decreased from about 4 in 1994 to about 1 in 2014. However, in 2015, it was elevated compared to the average rate in 2012–2014 in 11 members states out of 28 EU countries [5]. In South Korea, the number of accidents per 1000 employees was 7.3 in 2000, 6.9 in 2010, and 5.0 in 2015. The number of fatal injuries also declined from 15.6 per 100,000 in 2000 to 10.1 in 2015, but it is still high compared to that in the European countries [6,7].

Factors known to be associated with occupational injuries and accidents are individual (age, sex, race, working period, education, income, personality, smoking, drinking), job-related (occupation, employment type, working time, workload, job satisfaction, job stress), organization-related (workgroup size, management support, workplace safety status), and workplace-related (physical, musculoskeletal, chemical, and biological risk factors) [8,9,10,11]. In this study, we focus on the physical and musculoskeletal factors that workers are frequently exposed to, and which directly affect their health and safety in the workplace. Typical physical factors include vibration, noise, and abnormal temperatures (both high and low). Exposure to high levels of vibration can cause hand-arm vibration syndrome (HAVS), a typical occupational disease. HAVS causes pain and a decrease in sensitivity, agility, and muscle strength, which in turn can increase the likelihood of injury and accidents [12]. Excessive exposure to noise can cause hearing loss, which can then lead to secondary communication problems. It also increases fatigue and decreases concentration [13], both of which have been reported to increase the likelihood of occupational injuries and accidents [14,15,16]. Abnormal temperatures are also reported to increase occupational injury [17,18]. Exposure to high temperatures causes not only direct burns but also febrile illnesses such as heat stroke and heat exhaustion [19], which in turn can reduce work efficiency and increase the likelihood of accidents [20,21]. Exposure to low temperatures, on the other hand, has been shown to increase the morbidity of musculoskeletal disorders [22] and in severe cases, to hypothermia [17], leading to increased secondary occupational injuries and accidents. The typical musculoskeletal factors associated with occupational injuries and accidents are awkward posture, handling of heavy objects, and repetitive tasks [10,23], all of which result in musculoskeletal disorders [24] and lower physical abilities, thus increasing the risk of secondary occupational injuries and accidents.

Based on past studies, thorough management of the physical and musculoskeletal factors is necessary to reduce occupational injuries and accidents. However, it is not easy to manage these factors simultaneously with limited resources. Therefore, it is necessary to prioritize them based on different situations and manage them effectively. Very few studies have evaluated these risk factors in South Korea. In this study, we evaluate different physical and musculoskeletal risk factors by classifying the subjects based on gender and the size of their company (number of employees) to reduce occupational injuries and accidents in the workplace.

## 2. Materials and Methods

### 2.1. Study Population

This study was based on the data of the 4th Korean Working Condition Survey (KWCS) conducted by the Korea Occupational Safety and Health Research Institute (OSHRI) from June 2014 to September 2014. The KWCS was developed on the basis of the European Working Conditions Survey (EWCS); furthermore, its validity and reliability had been assured in a past study [25]. The survey subjects included wage workers, self-employed workers and business owners who were 15 years or older. The Korea OSHRI hired professional researchers to conduct the survey and trained them on interviewing methods; such as paper and pen interview (PAPI) using paper questionnaires and computer-assisted personal interview (CAPI). Next, a total of 50,007 subjects were interviewed by the researchers in 17 cities over four months.

In this study, we excluded 5654 subjects with missing values, 1208 subjects whose employment type was not clear, and 28,764 subjects who were managers, experts, and office, sales or service workers all of who are at low risk of occupational injuries and accidents. Finally, we included 14,381 individuals (9776 men and 4605 women) in the study.

### 2.2. Survey

From the 4th KWCS the following data were assessed: age, gender, work period, working hours per week, education, occupation, employment type, monthly income, company size (number of employees), physical factors (vibration, noise, abnormal temperature), musculoskeletal factors (awkward posture, handling of heavy objects, repetitive task), occupational injuries, and occupational accidents.

#### 2.2.1. General Characteristics

Based on their level of education, they were categorized as “middle school graduate or below,” “high school graduate,” and “college or above.” Based on their occupation, they were grouped as “agricultural or fishery workers,” “engineers,” “operators or assembly workers,” and “others” which included military man and simple worker (guard, sweeper, delivery- man, and driver). The types of employment were “self-employed,” “permanent worker,” “temporary worker,” and “others” which included the “unpaid family member” and “unknown.” Monthly income was classified as “under 1000”, “1000–1990”, “2000–2990”, “3000–3990”, “4000 or above” thousand won and “no answer or unknown.” Based on the number of employees, the companies were divided into “under 50” and “50 or above”, with the former considered to be a risk to workers’ safety and health management because they are under no obligation to appoint a safety and health manager in South Korea [26].

#### 2.2.2. Occupational Injuries and Accident, Physical Factors, and Musculoskeletal Factors

Occupational injuries and accidents were defined as experiences of injury or accident. If subjects answered with “Yes” to the question “Over the last 12 months, did you experience any injury or accident at your workplace?” or “Did you experience any injury or accident in your current job?”, they were classified into the case group; otherwise, they were classified into the control group.

Exposure to physical and musculoskeletal factor was identified in response to the following questions on exposure time:

(1) Vibration: Are you exposed to vibration caused by hand tools, machinery, etc. at your workplace?
(2) Noise: Are you exposed to such severe noise levels that you have to raise your voice while speaking to others at your workplace?
(3) Abnormal temperature: Are you exposed to high temperatures that cause sweating at your workplace even when you are not working? or are you exposed to low temperatures (indoors or outdoors) at your workplace?
(4) Awkward posture: Are you exposed to tired or painful postures at your workplace?
(5) Handling of heavy objects: Do you drag, push, or move heavy objects at your workplace?
(6) Repetitive task: Do you perform repetitive hand or arm movements at your workplace?

The questions on time of exposure to physical and musculoskeletal factors had seven possible responses: entire working time, almost the whole working time, 3/4 of the working time, 1/2 of the working time, 1/4 of the working time, almost never, and never. To prevent overestimation of exposure, we defined “presence of exposure” as exposure for more than 1/2 of the working time. While the first four responses were classified as “presence of exposure”, the remaining three were classified as “absence of exposure.”

### 2.3. Statistical Analysis

We compared the incidence of occupational injuries and accidents based on age, work period, and working hours per week using the Student’s t-test. We also used the chi-square test to make comparisons based on gender, education, occupation, employment type, monthly income, and the company size. Additionally, the influence of physical and musculoskeletal factors was compared using the chi-square test by categorizing the subjects based on gender and company size.

Considering gender and company size together, the subjects were reclassified into four subgroups (“under 50, male”, “under 50, female”, “50 or above, male”, “50 or above, female”) to compare the risk of occupational injuries and accidents based on physical or musculoskeletal factors. In the case of simultaneous exposure of various risk factors, determine which risk factors affect occupational injuries and accidents may be difficult. Therefore, only the case of single exposure, except multiple exposure of risk factors, was analyzed in the same category. Logistic regression analysis was performed to compare the odds ratio (OR) based on the presence or absence of each physical factor. Also, multiple logistic regression analysis was performed to compare the adjusted odds ratio (aOR) for age, work period, working hours per week, education, occupation, employment type, and monthly income. The same analysis was also performed for musculoskeletal factors.

The KWCS sample design used a secondary probability proportion stratified cluster sample survey (first extraction, extract stratified survey districts; second extraction, extract household and household members). In this sampling process, bias may arise due to differences in the population structure of the survey districts. Therefore, the KWCS provided a weight to adjust for this bias and recommended applying weighting adjustment in data analyses. Weighting adjustment is the process by which the sample is made similar to the population structure of South Korea. Therefore, we applied weighting adjustment in our analyses of the data. This study used SPSS 24.0 (IBM SPSS Inc., Chicago, IL, USA) to analyze all data, and *p*-values less than 0.05 were considered statistically significant.

## 3. Results

Finally, after adjusting for weight 12,984 subjects (8923 men and 4061 women) were analyzed, and 1147 of them experienced occupational injuries and accidents. Subjects who had suffered an occupational injury or accident were of a significantly higher age, with longer work period, and put in more working hours per week than those who did not have an occupational injury or accident. The incidence of occupational injuries and accidents was higher among men and tended to decline with an increase in education level. Based on occupation and employment type, it was the highest among the agricultural or fishery workers and the self-employed. The incidence also rose with an increase in monthly income and was higher in companies with under 50 employees (Table 1). An analysis of the risk factors revealed that in men the occupational injuries and accidents were related to all the physical and musculoskeletal factors, while in women, they were related to all the musculoskeletal factors, vibration and abnormal temperatures (Table 2). Based on the company size, while in the “under 50” companies occupational injuries and accidents were associated with all the physical factors, in the “50 or above” companies they were associated with only vibration, noise and awkward posture (Table 3).

In the “under 50, male” category, the OR for vibration and abnormal temperatures showed a significant relation [OR of vibration: 2.00 (95% CI 1.57–2.57); OR of abnormal temperature: 1.56 (95% CI 1.24–1.98) with occupational injuries and accidents, but after adjusting for co-variants, only vibration retained a significant relation [aOR of vibration: 2.05 (95% CI 1.57–2.66)]. However, all musculoskeletal factors were significantly associated before [OR of awkward posture: 2.15 (95% CI 1.66–2.78); OR of handling of heavy objects: 2.22 (95% CI 1.66–2.97); OR of repetitive task: 1.65 (95% CI 1.14–2.38)] and after [aOR of awkward posture: 2.01 (95% CI 1.60–2.53); aOR of handling of heavy objects: 2.00 (95% CI 1.52–2.65); aOR of repetitive task: 1.90 (95% CI 1.39–2.59)] adjusting for co-variants. In the “50 or above, male” category, among the physical factors, both vibration [OR: 3.07 (95% CI 1.85–5.10) and aOR: 2.96 (95% CI 1.74–5.02)] and noise [OR: 4.34 (95% CI 1.97–9.57) and aOR: 4.96 (95% CI 2.16–11.36)] were significantly related to the incidence of occupational injuries and accidents. Among musculoskeletal factors, awkward posture was significantly related to the incidence of occupational injuries and accidents [OR: 2.26 (95% CI 1.42–3.60), aOR: 2.30 (95% CI 1.43–3.70)]. In the “under 50, female” category, vibration and abnormal temperatures among physical factors were significantly related [OR for vibration: 3.33 (95% CI 2.14–5.19); OR for abnormal temperature: 2.06 (95% CI 1.42–2.99)] to occupational injuries and accidents.

However, after adjusting for co-variants, only vibration remained a significant factor [aOR of vibration: 3.64 (95% CI 2.20–6.00)]. In the “50 and above, female” category, there was no significant association between physical/musculoskeletal factors and occupational injuries and accidents (Table 4).

## 4. Discussion

Occupational injuries and accidents are serious problems that not only lower the quality of workers’ lives but can even be life-threatening [1]. Though several factors are known to be associated with their occurrence [8,9,10,11], it is not easy to manage all of them in the actual workplace. Therefore, in this study, we assessed the significance of different physical and musculoskeletal factors to enable their effective management to reduce the incidence of injuries and accidents in the workplace. Using data from the 4th KWCS conducted by the Korea OSHRI from June 2014 to September 2014, we stratified and analyzed subjects based on gender and company size.

Our findings show that in men, occupational injuries and accidents were associated with all the physical and musculoskeletal factors, while in women they were associated with all these factors except noise. Similarly, while in the “under 50” companies, occupational injuries and accidents were associated with all the physical and musculoskeletal factors, in the “50 or above” companies, they were associated with only vibration, noise, and awkward posture. These findings are in line with those of previous studies [8,9,27] and therefore, confirm that differences exist in the incidence of occupational injuries and accidents based on gender and workplace size in South Korea.

For further analysis, subjects were divided into four groups based on the combination of workplace size and gender, and included (1) under 50 and male, (2) under 50 and female, (3) 50 or above and male, and (4) 50 or above and female. After that, the OR and aOR for occupational injuries and accidents were calculated for the physical (vibration, noise, abnormal temperature) and musculoskeletal factors (awkward posture, handling of heavy objects, repetitive task). The analysis showed that among men, the risk of occupational injuries and accidents was associated with all the musculoskeletal factors and vibration in companies with “under 50” employees, while they were associated with only vibration, noise and awkward posture in companies with “50 or above” employees. On the other hand, among women, only vibration was found to increase the risk of occupational injuries and accidents in workplaces with “under 50” employees. Although earlier studies have analyzed factors that increase the risk of occupational injuries and accidents [8,9,10,11], our findings have important implications, since not much is known about the significance of these factors in different situations. Our results, therefore, demonstrate that the risk factors may differ based on the worker’s gender and the workplace size, which in turn helps in prioritizing them for the management of workplace safety.

It is known that the cost of management of work-related injuries and illnesses is very high. The International Labor Organization (ILO) estimated that an average of 4% of global GDP was spent on work-related injuries and illnesses [3]. In case of South Korea, 269,510 workers were recognized as having work-related injuries and illness in 2016. Of these, 222,577 were identified as having occupational accidents and thus, accounted for approximately 83% of the total cases. As a result, the insurance benefit paid for these cases amounted to approximately 2.85 billion dollars (3.3% of the GDP in 2016); this value accounts for 75% of the total cost of insurance benefits paid due to work-related injuries and illness [28]. To reduce these losses, it is necessary to find effective ways for reducing occupational injuries and accidents. Recently, studies based on past databases have been conducted to reduce occupational injuries and accidents [29,30]. However, practical ways with consideration of individual, job-related, organization, and workplace factors have rarely been investigated. Furthermore, only simple suggestions of principles to reduce occupational injuries and accidents have been put forward [31]. Therefore, it is still difficult to determine which measures should be taken in order to effectively reduce occupational injuries and accidents in a number of real-life situations. In this respect, it is necessary to confirm the importance of the results of this study and to provide information on safety management at the workplace that has practical applications in the field.

Although we obtained meaningful results, our study has some limitations. First, as this was a cross-sectional study, we could not explain any causal relationships. Second, because the data analyzed were limited to those obtained from the KWCS, we could not consider any additional risk factors. Third, there was a possibility that the accuracy of the analysis may have been lowered due to the differences in the number of subjects in various subgroups as a result of the stratification in several steps. Fourth, because the survey was based on the questionnaire, the risk factors were identified only from the subjective viewpoint of the subjects, and determining the exact size of the company was difficult.

However, the findings of this study are highly reliable because they are based on the KWCS data that represents the Korean workers and validity and reliability of the KWCS has been assured [25]. Also, these results provide directions for the management of risk factors based on gender and workplace size to reduce occupational injuries and accidents in the actual workplace. Finally, to our knowledge, a study of this type has not yet been performed in South Korea, which makes it a significant one.

## 5. Conclusions

Workers are exposed to various risk factors and are likely to suffer injuries and accidents. Although the overall incidence of occupational injuries and accidents has decreased, it is still a continuing problem which results in a deterioration of the workers’ quality of life and an increase in various related costs. However, in spite of these problems being confirmed, there have been only a few studies on how to manage and prioritize them to prevent their occurrence. Although this study focusses only on the problem of risk factor management according to gender and company size, we believe that continuing research in this direction will help reduce the occurrence of occupational injuries and accidents in the future.

## Figures and Tables

**Table 1:** General characteristics of the study subjects.

Variables		Occupational Injuries and Accidents		*p*-Value
		No (*n* = 11,837)	Yes (*n* = 1147)	
Age (years)		53.1 ± 14.1	54.8 ± 13.8	<0.001
Work period (years)		10.7 ± 12.1	18.0 ± 16.2	<0.001
Working hours per week (hours)		44.8 ± 16.1	48.3 ± 13.8	<0.001
Gender	Male	7990 (89.5)	934 (10.5)	<0.001
	Female	3847 (94.7)	214 (5.3)	
Education	Middle school graduate or below	4045 (90.1)	442 (9.9)	0.008
	High school graduate	5940 (91.5)	549 (8.5)	
	College or above	1853 (92.2)	156 (7.8)	
Occupation	Agricultural or fishery worker	1217 (80.8)	290 (19.2)	<0.001
	Engineer	3535 (90.9)	356 (9.1)	
	Operator or assembly worker	2263 (91.4)	214 (8.6)	
	Others ^†^	4823 (94.4)	288 (5.6)	
Employment type	Self-employed	2710 (85.2)	472 (14.8)	<0.001
	Permanent worker	5697 (92.8)	445 (7.2)	
	Temporary worker	3430 (93.7)	231 (6.3)	
Monthly income (thousand Won ^‡^)	Under 1000	2768 (92.2)	233 (7.8)	<0.001
	1000–1990	4308 (92.2)	365 (7.8)	
	2000–2990	2784 (90.8)	282 (9.2)	
	3000–3990	1236 (88.6)	159 (11.4)	
	4000 or above	552 (85.8)	91 (14.2)	
	No answer or unknown	189 (91.3)	18 (8.7)	
Number of employees	Under 50	9637 (90.9)	970 (9.1)	0.010
	50 or above	2200 (92.5)	178 (7.5)	

^†^ Others: simple worker (guard, sweeper, delivery-man, driver, etc.), a military man; ^‡^ 1 thousand Won = about 0.9 USD; 1 thousand Won = about 0.8 EUR.

**Table 2:** Differences in the prevalence of occupational injuries and accidents among the two genders based on physical and musculoskeletal factors.

Gender	Variables			Occupational Injuries and Accidents		*p*-Value
				No	Yes	
Male	Physical factors	Vibration	No	5126 (91.9)	449 (8.1)	<0.001
			Yes	2863 (85.5)	484 (14.5)	
		Noise	No	6093 (90.6)	631 (9.4)	<0.001
			Yes	1897 (86.3)	302 (13.7)	
		Abnormal temperature ^†^	No	5309 (90.9)	534 (9.1)	<0.001
			Yes	2681 (87.0)	399 (13.0)	
	Musculoskeletal factors	Awkward posture	No	4466 (92.8)	345 (7.2)	<0.001
			Yes	3524 (85.7)	588 (14.3)	
		Handling of heavy objects	No	5229 (92.2)	440 (7.8)	<0.001
			Yes	2761 (84.8)	493 (15.2)	
		Repetitive task	No	6309 (90.8)	638 (9.2)	<0.001
			Yes	1680 (85.0)	296 (15.0)	
Female	Physical factors	Vibration	No	3119 (95.6)	145 (4.4)	<0.001
			Yes	728 (91.3)	69 (8.7)	
		Noise	No	3375 (94.7)	189 (5.3)	0.664
			Yes	472 (95.2)	24 (4.8)	
		Abnormal temperature	No	2905 (95.4)	141 (4.6)	0.002
			Yes	943 (92.8)	73 (7.2)	
	Musculoskeletal factors	Awkward posture	No	2062 (95.6)	94 (4.4)	0.006
			Yes	1785 (93.7)	120 (6.3)	
		Handling of heavy objects	No	3037 (95.3)	151 (4.7)	0.004
			Yes	810 (92.8)	63 (7.2)	
		Repetitive task	No	2876 (95.6)	133 (4.4)	<0.001
			Yes	971 (92.3)	81 (7.7)	

^†^ Abnormal temperature: exposure to a high or low temperature in the workplace.

**Table 3:** Differences in the prevalence of occupational injuries and accidents in workplaces of different sizes based on physical and musculoskeletal factors.

Number of Employees	Variables			Occupational Injuries and Accidents		*p*-Value
				No	Yes	
Under 50	Physical factors	Vibration	No	6953 (93.1)	519 (6.9)	<0.001
			Yes	2685 (85.6)	450 (14.4)	
		Noise	No	7914 (91.7)	721 (8.3)	<0.001
			Yes	1724 (87.4)	249 (12.6)	
		Abnormal temperature ^†^	No	6654 (92.2)	560 (7.8)	<0.001
			Yes	2983 (87.9)	409 (12.1)	
	Musculoskeletal factors	Awkward posture	No	5278 (93.5)	369 (6.5)	<0.001
			Yes	4360 (87.9)	600 (12.1)	
		Handling of heavy objects	No	6614 (93.4)	466 (6.6)	<0.001
			Yes	3024 (85.7)	504 (14.3)	
		Repetitive task	No	7629 (92.2)	642 (7.8)	<0.001
			Yes	2009 (86.0)	327 (14.0)	
50 or above	Physical factors	Vibration	No	1292 (94.5)	75 (5.5)	<0.001
			Yes	907 (89.8)	103 (10.2)	
		Noise	No	1554 (94.0)	100 (6.0)	<0.001
			Yes	646 (89.3)	77 (10.7)	
		Abnormal temperature	No	1560 (93.1)	115 (6.9)	0.076
			Yes	640 (91.0)	63 (9.0)	
	Musculoskeletal factors	Awkward posture	No	1251 (94.7)	70 (5.3)	<0.001
			Yes	949 (89.8)	108 (10.2)	
		Handling of heavy objects	No	1652 (93.0)	125 (7.0)	0.184
			Yes	547 (91.3)	52 (8.7)	
		Repetitive task	No	1557 (92.4)	128 (7.6)	0.748
			Yes	643 (92.8)	50 (7.2)	

^†^ Abnormal temperature: exposure to a high or low temperature in the workplace.

**Table 4:** OR and aOR for occupational injuries and accidents based on physical and musculoskeletal factors according to gender, and company size.

Number of Employees	Variables		Male					Female				
			Number	OR ^†^ (95% CI)		aOR ^‡^ (95% CI)		Number	OR ^†^ (95% CI)		aOR ^‡^ (95% CI)	
Under 50	Physical factors	No	3439	1.00		1.00		2205	1.00		1.00	
		Vibration	693	2.00	(1.57–2.57)	2.05	(1.57–2.66)	250	3.33	(2.14–5.19)	3.64	(2.20–6.00)
		Noise	62	0.77	(0.27–2.21)	0.77	(0.27–2.25)	34	0.59	(0.06–5.71)	0.39	(0.04–3.99)
		Abnormal temperature	961	1.56	(1.24–1.98)	1.24	(0.97–1.58)	605	2.06	(1.42–2.99)	1.19	(0.80–1.78)
	Musculoskeletal factors	No	2462	1.00		1.00		1328	1.00		1.00	
		Awkward posture	1074	2.15	(1.66–2.78)	2.01	(1.60–2.53)	771	1.01	(0.64–1.58)	0.86	(0.54–1.37)
		Handling of heavy objects	698	2.22	(1.66–2.97)	2.00	(1.52–2.65)	159	1.04	(0.46–2.37)	0.72	(0.31–1.68)
		Repetitive task	474	1.65	(1.14–2.38)	1.90	(1.39–2.59)	319	1.54	(0.90–2.63)	1.68	(0.96–2.95)
50 or above	Physical factors	No	806	1.00		1.00		299	1.00		1.00	
		Vibration	211	3.07	(1.85–5.10)	2.96	(1.74–5.02)	61	0.98	(0.22–4.31)	1.12	(0.24–5.22)
		Noise	49	4.34	(1.97–9.57)	4.96	(2.16–11.36)	8	4.79	(0.62–36.88)	3.39	(0.38–30.05)
		Abnormal temperature	115	0.98	(0.40–2.44)	0.99	(0.39–2.50)	51	0.52	(0.06–4.18)	0.63	(0.07–5.48)
	Musculoskeletal factors	No	653	1.00		1.00		181	1.00		1.00	
		Awkward posture	328	2.26	(1.42–3.60)	2.30	(1.43–3.70)	98	2.58	(0.83–7.97)	2.15	(0.64–7.17)
		Handling of heavy objects	122	0.82	(0.34–2.00)	0.98	(0.40–2.41)	20	-	-	-	-
		Repetitive task	216	1.17	(0.63–2.19)	1.21	(0.64–2.27)	94	1.19	(0.30–4.69)	0.76	(0.18–3.20)

^†^ Odds ratio was calculated by logistic regression analysis; ^‡^ Adjusted odds ratio was calculated by multiple logistic regression analysis after adjusting for age, work period, working hours per week, education, occupation, employment type, and monthly income.
